# *De Novo* Transcriptome Sequencing of *Oryza officinalis* Wall ex Watt to Identify Disease-Resistance Genes

**DOI:** 10.3390/ijms161226178

**Published:** 2015-12-10

**Authors:** Bin He, Yinghong Gu, Xiang Tao, Xiaojie Cheng, Changhe Wei, Jian Fu, Zaiquan Cheng, Yizheng Zhang

**Affiliations:** 1 Key Laboratory of Bio-Resources and Eco-Environment, Ministry of Education, Sichuan Key Laboratory of Molecular Biology and Biotechnology, College of Life Sciences, Sichuan University, Chengdu 610064, China; hebin.li@foxmail.com (B.H.); yinghonggu@gmail.com (Y.G.); xiaoxiaojie1991@foxmail.com (X.C.); hantanheying@126.com (C.W.); 2 Chengdu Institute of Biology, Chinese Academy of Sciences, Chengdu 610041, China; taoxiang180@gmail.com; 3 Biotechnology & Genetic Resources Institute, Yunnan Academy of Agricultural Sciences, Kunming 650223, China; fugein@aliyun.com

**Keywords:** *Oryza officinalis*, transcriptome, *de novo* assembly, disease-resistant genes

## Abstract

*Oryza officinalis* Wall ex Watt is one of the most important wild relatives of cultivated rice and exhibits high resistance to many diseases. It has been used as a source of genes for introgression into cultivated rice. However, there are limited genomic resources and little genetic information publicly reported for this species. To better understand the pathways and factors involved in disease resistance and accelerating the process of rice breeding, we carried out a *de novo* transcriptome sequencing of *O. officinalis*. In this research, 137,229 contigs were obtained ranging from 200 to 19,214 bp with an N50 of 2331 bp through *de novo* assembly of leaves, stems and roots in *O. officinalis* using an Illumina HiSeq 2000 platform. Based on sequence similarity searches against a non-redundant protein database, a total of 88,249 contigs were annotated with gene descriptions and 75,589 transcripts were further assigned to GO terms. Candidate genes for plant–pathogen interaction and plant hormones regulation pathways involved in disease-resistance were identified. Further analyses of gene expression profiles showed that the majority of genes related to disease resistance were all expressed in the three tissues. In addition, there are two kinds of rice bacterial blight-resistant genes in *O. officinalis*, including two *Xa1* genes and three *Xa26* genes. All 2 *Xa1* genes showed the highest expression level in stem, whereas one of *Xa26* was expressed dominantly in leaf and other 2 *Xa26* genes displayed low expression level in all three tissues. This transcriptomic database provides an opportunity for identifying the genes involved in disease-resistance and will provide a basis for studying functional genomics of *O. officinalis* and genetic improvement of cultivated rice in the future.

## 1. Introduction

*Oryza officinalis* Wall ex Watt (CC, 2n=2x=24) is one of the important wild species in *Oryza* genus. It is one of the three wild species indigenous in China [1]. It has reservoirs of many useful genes for rice breeding, such as resistance to blast, bacterial blight (BB), brown planthopper (BPH), and white backed planthopper (WBPH) [2,3,4,5]. It can also tolerate drought, cold, and other abiotic stresses [6,7]. Through interspecific hybridization and backcrossing between *O. officinalis* and *Oryza sativa*, a number of resistance genes have been introduced into cultivars and some varieties have been released for commercial cultivation. In 2007, several introgression lines from an *O. sativa* × *O. officinalis* cross were produced to enhance blast resistance ability by Jena and Khush [8]. One of the most successful examples of the transfer of genes from *O. officinalis* is that of the resistance genes to brown planthopper (BPH). Four BPH resistance genes, *Bph10*, *Bph18*, *bph11*, and *bph12*, were transferred from *O. officinalis* to cultivated rice. Four breeding lines were released as varieties (MTL95, MTL98, MTL103, and MTL110) for commercial cultivation in Mekong Delta, Vietnam [9]. In addition, Huang *et al.* also transferred the BPH resistance gene from *O. officinalis* into Zhensheng 97B to improve disease resistance [10]. However, the present understanding of the disease resistance mechanism and related disease-resistance genes in *O. officinalis* is limited.

Successful implementation of a defense response requires that plants respond to a pathogen rapidly and accurately. Signaling events in the plant–pathogen interaction are critical for the process of pathogen recognition and lead to hypersensitive response (HR). During the process, a first layer is based on the amazingly sensitive perception of pathogen/microbe–associated molecular patterns (PAMPs or MAMPs) through pattern recognition receptors (PRRs) at the plant’s cell surface. The second layer of defense is called effector-triggered immunity (ETI), in which plants, in turn, can recognize such effectors through additional receptors, particularly nucleotide-binding leucine-rich repeat (NB-LRR) proteins [11]. Besides, evidence has accumulated over the past few years to indicate that plant hormones, such as salicylic acid (SA), jasmonic acid (JA) and abscisic acid (ABA), also play an important regulatory role in the plant immune response, such as determining the recognition of PAMPs, mediating system-acquired resistance and activating the hypersensitive response. Related genes on the plant–pathogen interaction and the plant hormones regulation were reported in some organisms [12].

More recently, genome-wide approaches have become valuable tools in characterizing gene expression and elucidating the genetic networks of disease resistance at a global level [13]. In the past, the draft genome sequence of the *O. sativa* ssp. *indica* cv. 9311 and the full genome sequence of *O. sativa* ssp. *japonica* cv. Nipponbare were completed through a whole-genome shotgun sequencing approach and a map-based sequencing strategy, respectively [14,15]. Even so, the cost of genome sequencing is too high to afford. Therefore, much effort was involved in rice EST projects. Approximately 1,249,110 ESTs and more than 50,000 FLcDNA sequences of cultivated rice are currently available in public databases. However, the genomic studies of rice wild relatives are still in their infancy with the exception of the generation of 1888 leaf FLcDNAs from the *O. rufipogon* (AA genome), 5211 leaf ESTs from the *O. minuta* (BBCC genome), 68,132 leaf unigenes from *O. officinalis* (CC genome) and 71,367 root unique sequences from the *O. longistaminata* (AA genome) [7,16,17,18]. Furthermore, genes that might be expressed in different organs are underrepresented in EST studies.

To further explore the *O. officinalis* transcriptome, we employed RNA-seq for three tissues, *i.e.*, leaves, stems and roots. In this study, we provide a more comprehensive transcriptome of *O. officinalis* using the next-generation sequencing platform Illumina. The large EST dataset will remarkably enlarge genomic resources of *O. officinalis* available in the public database. The transcriptome data have led to the identification of a number of candidate genes and functional elements determining plant–pathogen interaction and plant hormone regulation pathways associated with diseases resistance, which are of great importance to efforts for genetic improvement of the cultivated rice.

## 2. Results and Discussion

### 2.1. De Novo Assembly and Quality Assessment

To develop an overview of the *O. officinalis* transcriptome and to obtain the representative transcripts, three tissues representing various vegetative tissues, including leaves, stems and roots, were harvested for RNA isolation. Following the Illumina manufacturer’s instructions, the shotgun libraries were constructed and used for sequencing with the Illumina High-Seq 2000 platform. A total of 54,941,770, 54,282,656 and 53,625,698 raw reads with a length of 90 bp were generated from *O. officinalis* leaves, stems and roots transcripts, respectively. After quality checks and trimming of the adapter, the assembly strategy of single-assembler multiple-parameters (SAMP) from Oases was employed to assemble the transcriptome on the basis of our previous study [19]. Finally, a total of 137,229 contigs were generated ranging from 200 to 19,214 bp with an N50 of 2331 bp (Table 1). The majority of the assemblies were over 500 bp in size (71%) and 28% of them were longer than 2000 bp.

**Table 1:** Summary of sequence assembly and function annotation of the *O. officinalis* transcriptome.

Items	Number of Sequences
**Assembly**	
Total number of contigs	137,299
Mean length (bp)	1459
Maximum contig length (bp)	19,214
N50 (bp)	2331
Number of contigs (≥1 kb)	72,521
**Annotation**	
Number of predicted ORFs	82,983
Number of predicted ORFs (≥900 bp)	53,239
Transcript BLASTx against NR	88,249
Transcript BLASTx against Pfam	79,310
Transcripts annotated with GO terms	75,589
Transcript annotations against KEGG	21,191

To assess how well the assembled sequences represent the actual transcriptome population, several approaches have been adopted. First, the transcriptome gene coverage was judged by comparison with the sequence information available for *O. officinalis* and phylogenetically related species, *O. sativa*. 58 of 69 complete protein-coding genes from *O. officinalis* and 429 of 523 proteins from *O. sativa* in the NCBI database were present in our assembled transcriptome. We then detected the transcript sequence quality and completeness through ORF prediction, since the optimal assembly results could produce long and complete ORFs, and as many as possible. A high proportion of the transcripts (82,983, 61%) could be detected in ORFs in which 53,239 (64%) ORFs are longer than 900 bp. Thirdly, all reads were mapped to the contigs for counting the utilization ratio. 92.52% of them were found to be aligned back to the contigs, indicating that almost all reads were utilized for the *de novo* assembly. In addition, the accuracy of the assembled contigs was up to 82%. On the basis of the above evaluation, the quality of the assembled transcriptome is good enough for functional annotation and further analysis.

### 2.2. Functional Annotation of O. officinalis Transcriptome

**Figure 1:**
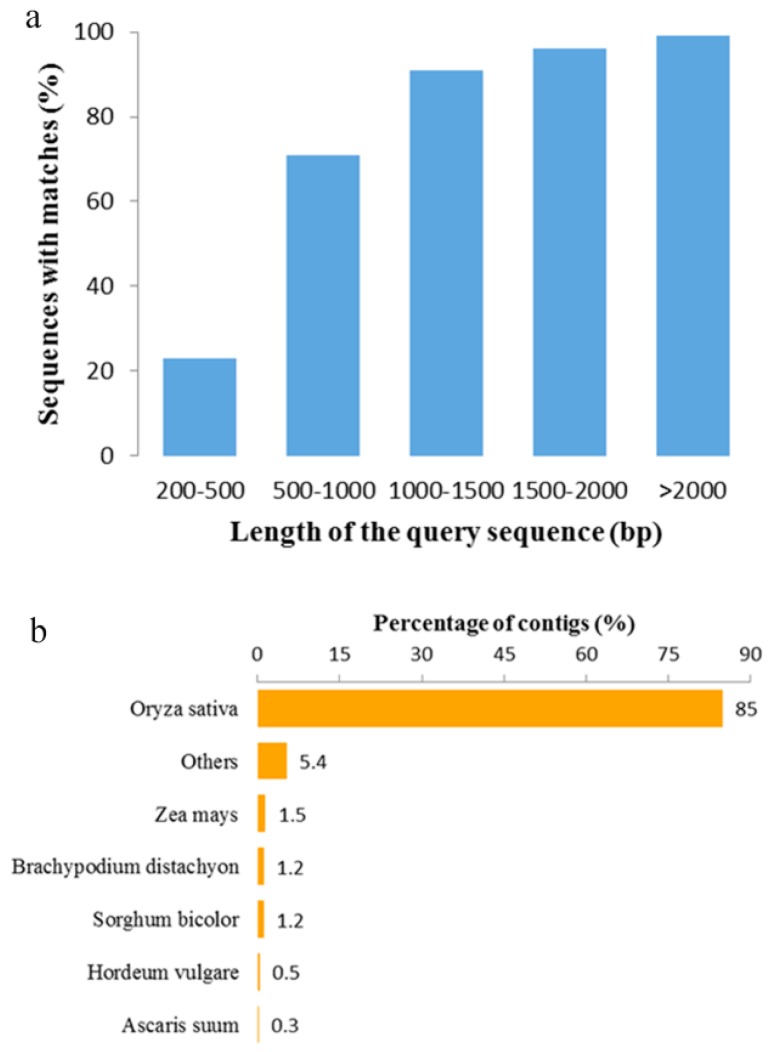
Characteristics of the homology search of contigs against the NR database. (**a**) Effects of query sequence length on percentage of significant matches. The cut-off value was set at 1.0 × 10^−5^. The proportion of sequences with matches in the NR database at NCBI is greater among the longer assembled sequences; (**b**) Species distribution is shown as the percentage of the total homologous sequences.

To predict and analyze the function of the transcripts of *O. officinalis*, all contig sequences were first aligned against the NCBI non-redundant (NR) protein database using BLASTx. A total of 88,249 significant BLAST top hits were returned with a cut-off *E*-value of 1 × 10^−5^ (64% of all transcripts; Table 1). As shown in the previous study, the length of contigs is crucial in determining the efficiency of BLAST searches [20]. Our results showed that 99% of the matching efficiency were observed for sequences longer than 2000 bp and 96% for those between 1500 to 2000 bp, whereas the matching efficiency decreased to about 71% for those ranging from 500 to 1000 bp and to 23% for sequences between 200 to 500 bp (Figure 1a). For species distribution, most of the sequences (85%) had the top matches with those from the *Oryza sativa*, followed by the Zea mays (1.5%), Brachypodium distachyon (1.2%), Sorghum bicolor (1.2%) and Hordeum vulgare (0.5%) (Figure 1b).

**Figure 2:**
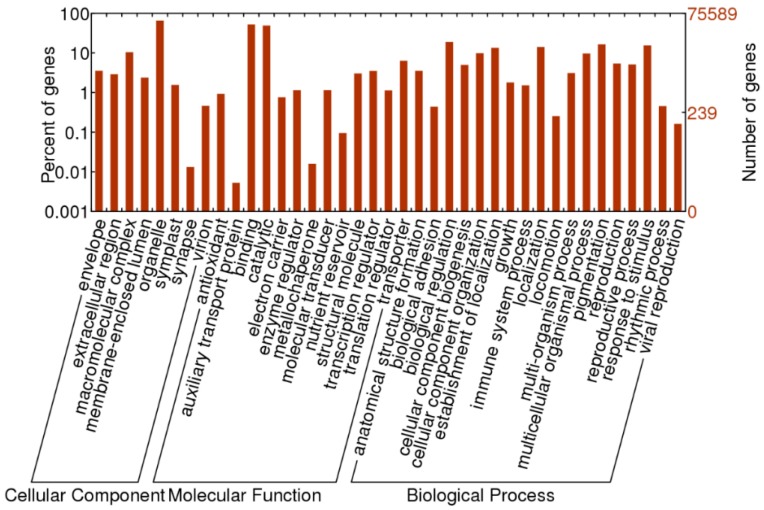
Gene Ontology classification of all *O. officinalis* transcripts.

To functionally classify the *O. officinalis* transcripts, sequences that had matches in NR databases achieved GO annotations with the Uniprot database. Of the 88,249 transcripts with NR annotation, a total of 75,589 transcripts were assigned to 369,110 GO terms. The distribution of GO terms for molecular functions, biological processes and cellular components is shown in Figure 2. Except the general categories, for the biological process classification, genes involved in the “biological regulation” (GO: 0065007) and “pigmentation” (GO: 0043473) were the most highly represented categories. For the molecular function classification, “binding functions” (GO: 0005488) was the most enriched GO term, followed by “catalytic functions” (GO: 0003824). For the cellular component, the major categories were “macromolecular complex” (GO: 0032991), “envelope” (GO: 0031975) and “organelle” (GO: 0043226).

### 2.3. Domain Prediction and KEGG Pathway Mapping

**Figure 3:**
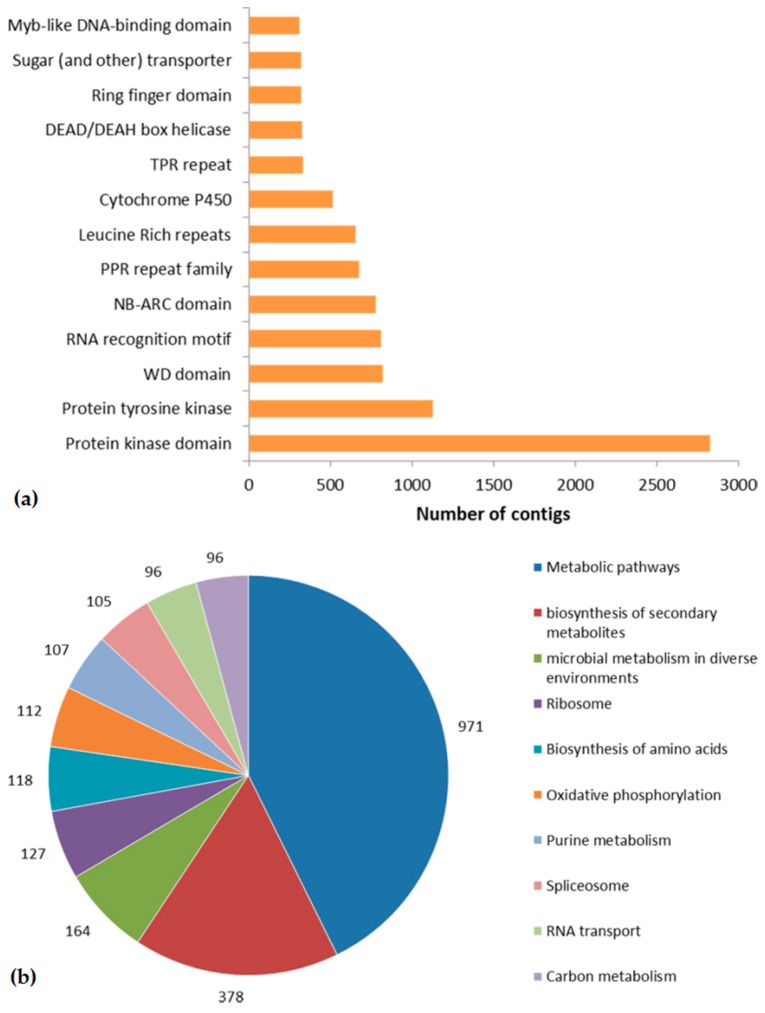
Domains prediction and KEGG classification of assembled contigs. (**a**) The 13 most abundant protein families in *O. officinalis*; (**b**) The top 10 pathways with the highest contig numbers.

Considering that the information of conserved domains within a gene was indicative of deducing genes’ function, we performed the annotation of potential domains inside the assembled transcripts. To facilitate this procedure, ORFs for each transcript were extracted using a set of programs included in the Trinity package (see Methods), and then all the transcripts with predicted ORFs were searched against the PFAM database using HMMER v3.0 locally. Overall, a total of 79,310 transcripts were categorized into 6384 domains/families. Based on the frequency of the occurrence of transcripts contained in each Pfam domain, the top thirteen abundant domains/families were ranked and listed in Figure 3a. Among these domains/families, the “protein kinasedomain” and its subclass “protein tyrosine kinase” play an important role in a multitude of cellular processes, including division, proliferation, apoptosis, and differentiation; proteins with the “leucine rich repeats” domain are recognized to be frequently involved in signaling, translation and protein-binding; and the “NB-ARC domain” has been reported to be shared by plant resistance gene products. Other protein families, such as “RNA recognition motif”, “WD domain, DEAD/DEAH box helicase, TPR repeat”, “cytochrome P450” and so on, which have some basic functions in plants, were also found in the top thirteen items on the list.

To fully identify the biological pathways that are active in *O. officinalis*, all the assembled sequences were assigned with Kyoto Encyclopedia of Genes and Genomes orthology (KO) identifiers using a KEGG Automatic Annotation Server (KAAS) with the single-directional best hit information method, and subsequently mapped to pathways and enzymes using the KEGG API. As a result, a total of 21,191 assembled sequences were found to be involved in 341 predicted KEGG metabolic pathways. The pathways with the most representation by the unique sequences were metabolic pathways (971 members), biosynthesis of secondary metabolites (378) and microbial metabolism in diverse environments (164). Figure 3b summarized the top 10 pathways with the most contigs. These results provide a valuable resource for investigating metabolic pathways in *O. officinalis*.

### 2.4. Genes Related to Disease Resistance

*O. officinalis* has been reported as a source of resistance to blast, bacterial blight, white-backed and brown planthoppers [4,5]. Therefore, it is very important to know the genes involved in plant resistance to disease in *O. officinalis* with regard to the following aspects: (1) the signaling events in the plant–pathogen interaction and their involvement in pathogen recognition; (2) the central role of plant hormones in the regulation of defense responses to pathogen; (3) and the specific examples of resistance genes, such as genes related to bacterial blight resistance.

#### 2.4.1. Signaling Events in the Plant–Pathogen/Insect Interaction

**Figure 4:**
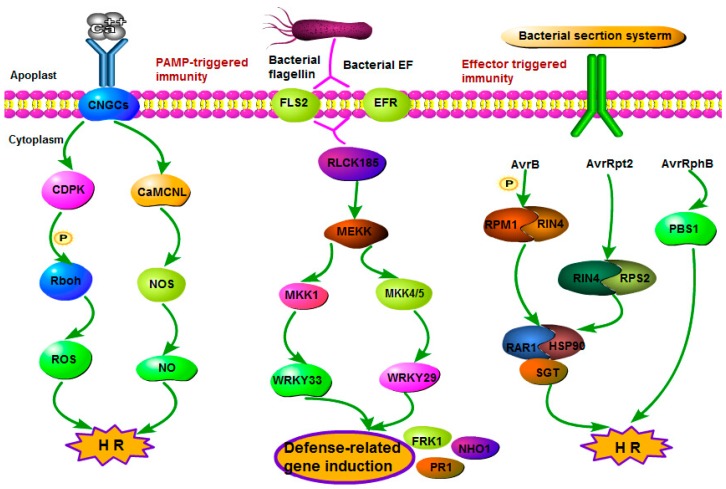
The plant–pathogen interaction pathway predicted in *O. officinalis*. This pathway is divided into two sub-pathway: PTI and ETI. The genes and transcription factors listed in this figure are all identified in *O. officinalis*.

Although the plant–pathogen interaction pathway has been known in *O. sativa*, existing knowledge on the pathways and enzymes involved in plant–pathogen interaction in wild rice, such as *O. officinalis*, is still limited. Figure 4 shows the plant–pathogen interaction pathway reconstructed based on the determined transcripts, and Table S1 lists all the transcripts.

All plants can recognize pathogen-associated molecular patterns (PAMPs) with multiple layers against invading pathogens. The primary response includes the perception of pathogens by cell-surface pattern-recognition receptors (PRRs) and is referred to as PAMP-triggered immunity (PTI). In addition to PRRs that recognize PAMPs, plants can also rely on immune receptors that recognize effectors or host plant targets manipulated by effectors resulting in effector-triggered immunity (ETI) [21]. Besides, the level of Ca^2+^ concentration is also a regulator for production of reactive oxygen species, cell wall reinforcement and hypersensitive response [22]. Based on the transcriptome annotation, in this study, we identified all of the genes encoding enzymes and proteins involved in the plant–pathogen interaction pathway in both *O. sativa* and *O. officinalis*. Moreover, three additional proteins are involved in the transcriptome of *O. officinalis*: WRKY transcription factor 29 (WRKY29: Contig_52053) and two R proteins (RAR: Contig_66753; FPK: Contig_15502), which may present novel genes expressed especially in *O. officinalis* but not in *O. sativa*. Recent studies revealed that OsPUB44 (Os06g10660), a rice ubiquitin E3 ligase, and OsRLCK185 (Os09g27890), a rice receptor-like cytoplasmic kinase, positively regulate immune responses [23]. In O. officinalis, we also identified one gene (Contig_47198) homologous to OsPUB44 with identity of 98% and two genes (Contig_23654 and Contig_26247) homologous to OsRLCK185 with identity of 89% and 84%. In addition, there is evidence that OsRLCK185 transmits the immune signal from OsCERK1 to the downstream mitogen-activated protein kinase (MAPK) cascade, while OsPUB44 functions in a different pathway than OsRLCK185 or downstream of the MAPK cascade [24]. Furthermore, some research has reported that LYP4 (Os09g27890) and LYP6 (Os06g10660) play a role in peptidoglycan perception and resistance against Xoo (*Xanthomonas oryzae* pv. *oryzae*) [25]. As expected, we identified one gene (Contig_60426) homologous to LYP4 with identity of 95% and one gene (Contig_59080) homologous to LYP6 with identity of 97% in *O. officinalis*. Although the previous research reported that transcription of these two genes could be induced rapidly upon exposure to bacterial pathogens or diverse MAMPs, their specific locations in PTI were not fully understood.

The results from expression analysis of genes related to plant–pathogen interaction in different tissues showed that CNGCs and RIN4 had a very low expression level in the three tissues. In addition, there were two genes, NOS and RPM1, only expressed in leaves. The other genes related to plant–pathogen interaction were all expressed in the three tissues (Table S1).

#### 2.4.2. Regulation of Defense Responses by Plant Hormones

To cope with destructive diseases, plants have evolved molecular mechanisms mediated by multiple, highly regulated and coordinated signaling networks that rely on plant hormones, including jasmonic acid (JA), salicylic acid (SA) and abscisic acid (ABA), as secondary messengers. 

**Figure 5:**
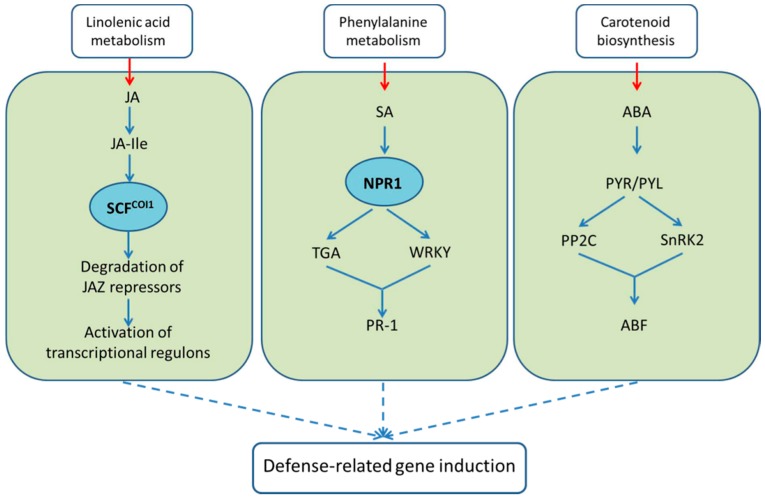
Genes of *O. officinalis* involved in the three plant hormones regulation pathways. The genes and transcription factors listed in this figure were all identified in *O. officinalis*.

Reviews of SA biosynthesis and signal transduction mechanisms has discussed its important regulatory role in mediating system-acquired resistance, activating the hypersensitive response and recognition of PAMPs, as well as activation of ETI [26]. In addition, when inflicted by chewing insects or mechanical damage, JA would be accumulated rapidly (<30 min) at the site of wounding [27]. In *O. sativa*, the routes of JA metabolism that modulate plant responses to disease are as follows: (1) Once JA have been synthesized through linolenic acid metabolism, it formed jasmonoyl–isoleucine (JA–Ile) and other JA–amino acid conjugates (JACs) through jasmonate resistance (JAR) and related conjugating enzymes; (2) the signal transduction of amino acid-conjugated forms of JA resulted in the formation of a coronatine-insensitive1 (COI1)–JA–JAZ ternary complex, a crucial node in downstream signaling pathways of JA, in which JAZs are then ubiquitinated and degraded; (3) rapid resynthesis of JAZ repressors presumably provides a mechanism to the activation of transcriptional regulons and potentially cell-damaging defensive processes. In the annotated transcriptome of *O. officinalis*, all of the key enzymes and proteins involved in the JA signal transduction were identified (Figure 5 and Table S2).

On the other hand, salicylic acid is also a key regulator of plant defense responses. In plants, the application of SA or SA analogs results in pathogenesis-related (PR) gene expression and then enhances disease resistance [28,29]. Firstly, accumulation of SA from phenylalanine metabolism induces a change in cellular redox potential triggering the reduction of non-expressed pathogenesis-related1 (NPR1) to active monomers which are then translocated to the nucleus and interacted with TGA transcription factors. Then, the binding of TGA factors to SA-responsive elements in the promoters of PR genes is stimulated by these interactions, and the subsequent transcriptional reprogramming leads to the activation of systemic acquired resistance (SAR). As proved in rice, the overexpression in rice of NPR1 confers enhanced resistance to *Xanthomonas Oryzae* pv. *Oryzae* (*Xoo*). In addition, recent studies revealed that OsWRKY45 plays a crucial role in NPR1-independent SA signaling in cultivated rice, and two allelic genes, OsWRKY45-1 (from japonica) and OsWRKY45-2 (from indica), which encode proteins with a 10-amino acid difference, play opposite roles in resistance against *Xoo* [30,31]. Here we identified one gene (Contig_52040) homologous to OsWRKY45. When comparing with the two allelic genes, OsWRKY45-1 (GenBank: GQ331932) and OsWRKY45-2 (GQ331927), OsWRKY45 in *O. officinalis* showed a difference with OsWRKY45-1 (from japonica) and OsWRKY45-2 (from indica) (Figure S1). Therefore, we inferred that OsWRKY45 may express in another allelic form neither OsWRKY45-1 nor OsWRKY45-2 in *O. officinalis*. Furthermore, the NPR1, TGA and PR1 from the transcriptome of *O. officinalis* involved in the SA signal transduction pathway were identified (Figure 5 and Table S2).

Abscisic acid (ABA) is a critical signaling molecule to activate disease resistance in different plant species through regulating various aspects of plant growth and development [32]. Hence, the biological and agricultural importance of ABA has led to increasing attention regarding its mechanism of signal transduction, and many putative signal transducers have been reported [33]. The PYR/PYL family proteins inhibit protein phosphatase 2C (PP2C) in an ABA-dependent manner as ABA receptors. Subsequently, PP2C interacts with a protein kinase, SnRK2, which is activated by osmotic stress or ABA and positively regulates the ABA response in various tissues. Furthermore, this complex stimulates the expression of ABA responsive element binding factor (ABF), which encodes a basic leucine zipper transcription factor. Finally, the expression of ABF enhanced resistance to necrotrophic pathogens. There is overwhelming evidence that high ABA concentrations interfere with resistance against pathogens controlled by the SA signaling pathway. Likewise, the genes related to SA signaling pathway were all detected in our *O. officinalis* transcriptome (Figure 5 and Table S2).

To assess the expression level of genes that are related to the regulation of defense responses by plant hormones (four for JA regulation, three for SA regulation and four for ABA regulation), differential expression analysis in three tissues was performed. From the results, JAR showed a very low expression level in the three tissues, while NPR1 displayed a high expression level. In addition, the differential expression analysis revealed that almost all of these genes related to the regulation of defense responses by plant hormones displayed a high expression level in three tissues, except JAZ, which was not expressed in leaves, and SnRK2, which did not exist in stems (Table S2). The results may reveal that plant hormones play important roles in defense responses and their regulation was not restricted to one tissue.

#### 2.4.3. Genes Related to Bacterial Blight Resistance

Bacterial blight, caused by Xanthomonas *Oryzae* pv. *Oryzae* (*Xoo*), is a devastating disease affecting rice worldwide. So far, seven bacterial blight resistance genes have been cloned and sequenced (*Xa1*, *xa5*, *xa13*, *Xa21*, *Xa23*, *Xa26* and *Xa27*) [34]. It is known that wild rice *O. officinalis* is resistant to bacterial blight. Therefore, we also identified the contigs involved in the bacterial blight resistance from *O. officinalis* transcripts. However, there were only 2 types of bacterial blight-resistance genes (*Xa1* and *Xa26*) identified in *O. officinalis* transcriptome. Two contigs (Contig 13873, Contig 1227) were annotated as bacterial blight-resistance protein Xa1 (named OofXa1_a,b), while three contigs (Contig 67329, Contig 77980, Contig 79023) were annotated as bacterial blight-resistance protein Xa26 (named OofXa26_a,b,c). However, the sequence of OofXa26_a,b,c were not complete. The results from expression analysis in different tissues showed that both Xa1 genes had the highest expression level in stem, whereas OofXa26_a was expressed dominantly in leaf and the other two Xa26 genes displayed low expression levels in all the three tissues.

**Figure 6:**
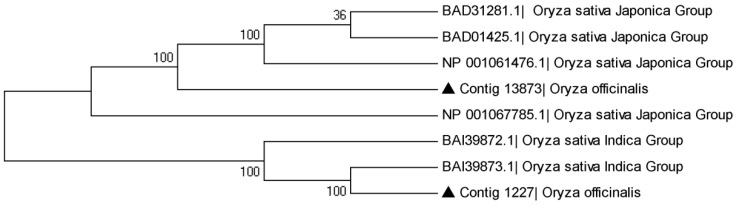
Phylogenetic relationship of the bacterial blight resistance protein Xa1 of *O. officinalis* with those of other species of the *Oryza* genus. Triangle symbol: OofXa1.

The phylogenetic relationship of Xa1 in *O. officinalis* transcriptome was also investigated. It was clearly shown that the phylogenetic tree is divided into two groups. Likewise, OofXa1_a and OofXa1_b were divided into two groups, in which OofXa1_a was clustered into the branch of *Oryza sativa Japonica* Group and OofXa1_b was clustered into the other branch (Figure 6).

## 3. Materials and Methods

### 3.1. Plant Materials and RNA Extraction

*O. officinalis* was planted at natural temperature and light in Yuanjiang, Yunnan Province of China. Three different tissues including mature leaves, stems and roots of *O. officinalis*, which were kindly provided by Zaiquan Cheng (Yunnan Academy of Agricultural Sciences, Kunming, China), were harvested when flowering. At least three independent biological replicates of each tissue sample were collected, snap-frozen immediately and then stored at −70 °C until processing. Total RNA was extracted with TRIzol Reagent (Invitrogen, Carlsbad, CA, USA) following the manufacturer’s instructions and treated with DNase I (Fermentas, Burlington, ON, Canada). After further extraction to remove the protein of DNase I, RNA integrity was confirmed using the Agilent 2100 Bioanalyzer (Agilent, Santa Clara, CA, USA) with a minimum integrity number value of 8.

### 3.2. cDNA Library Construction and Sequencing

The poly (A) + RNA (mRNA) was isolated and purified using Dynal oligo(dT) 25 magnetic beads (Invitrogen) following the manufacturer’s instructions. Followed by purification, the mRNA was fragmented into smaller pieces by the fragmentation buffer. Then the first-strand cDNA was synthesized using SuperScript III reverse transcriptase and N6 random hexamers based on the cleaved RNA fragments. Subsequently, second strand cDNA synthesis was performed by dNTPs, DNA Polymerase I and RNase H. The products were purified using AMPure XP beads and further processed by the ligation of adapters and an end repair. The purified products were enriched with PCR for preparing the final sequencing library. The quality control analysis of the cDNA library was performed by Agilent 2100 Bioanalyzer. The cDNA library was sequenced from both 5’ and 3’ ends using the Illumina HiSeq 2000 platform (Illumina, Dedham, MA, USA) according to the manufacturer’s protocol. Then the Illumina sequencing by-synthesis, image analysis and base-calling procedures were used to obtain paired-end (PE) read sequence information and base-calling quality values, in which 90 bp PE reads were obtained [35].

### 3.3. Data-Preprocessing, De Novo Assembly and Assembly Assessment

The raw reads were first filtered to remove adaptors, poly-A tails and contaminants using the FASTX tool kit (v 0.0.13, Gordon, LongIsland, NY, USA). Short (length <25 bp) reads and Low-quality (phred score <20) were trimmed by SolexaQA package (-l 25; −h 20) [36]. FASTQC (v 0.10.1, Andrews, Edinburgh, UK) was further used to detect the quality of the retained pair-end reads [37]. Clean reads were then *de novo* assembled using Single-Assembler Multiple-Parameters of Oases, which is an optimal assembly strategy for diploid organisms [19]. In order to evaluate the quality of assembly, four criterion were used: (i) gene coverage; (ii) transcript sequence quality and completeness; (iii) utilization ratio of reads; (iv) accuracy.

The gene coverage was judged by comparison with the sequence information available for *O. officinalis* and *O. sativa*, phylogenetically related species. There were 69 complete protein-coding genes from *O. officinalis* were all selected and 523 proteins from *O. sativa* in the NCBI database were randomly chosen as reference databases. The megablast and the common Perl analysis script were used to analyze the representation. Transcriptome quality was assessed by the best candidate coding sequence (CDS) for each contig using the TransDecoder program, which is included in the Trinity package [38]. To evaluate the utilization ratio of reads, all of the clean PE reads were aligned back to these contigs by using Bowtie2 (v2.0.0-beta5) program [39]. Finally, the accuracy was calculated with Acc = TP/(TP + FN) (TP = true positives, FN = false negatives), where Acc is accuracy, FN is the sum of all reference segment length that were not aligned, and TP is the sum of all aligned segment length (the overlap aligned regions were only calculated once) [40].

### 3.4. Gene Annotation and Differential Gene Expression Analysis

The assembled contigs were annotated by aligning against the NR databases using the BLASTX algorithm with a significance threshold of *E*-value of 10^−5^, retrieving proteins with the highest sequence similarity. To further annotate transcripts, the best Blastx hit from NR database for each transcript was submitted to the BLAST2GO platform to obtain GO terms [41]. WEGO (Web Gene Ontology Annotation Plot) is a useful tool for plotting GO annotation results and has been widely used in many important biological research projects. Therefore, we achieved GO classification through submitting GO annotation files in WEGO native format to WEGO [42]. Kyoto Encyclopedia of Genes and Genomes (KEGG) pathway mapping is the process to map molecular datasets, especially large-scale datasets in genomics, transcriptomics, proteomics, and metabolomics, to the KEGG pathway maps for biological interpretaion of higher-level systemic functions [43]. The assembled sequences were submitted to the online KEGG Automatic Annotation Server (KAAS) with single-directional best hit (SBH) method [44]. The output includes KO assignments and KEGG pathways that are involved with the KO assignments. For Pfam domain annotation, the predicted protein sequences were searched against the Pfam-A database (v27.0, Bateman, Hinxton, UK) using HMMER v3.0 (Johnson, MO, USA) locally [45].

Transcript abundances in *O. officinalis* leaf, stem and root tissues were estimated using edgeR in fragments per kilobase of transcript per million (FPKM) units. Genes that were differentially expressed among leaf, stem and root tissues were considered to be significantly different at an FDR (false discovery rate) <0.001, and an absolute value of log_2_fold-change (log_2_FC) ≥1, according to the method described by Audic and Claverie [46].

### 3.5. The Phylogenetic Relationship of Sequence

Another 6 genes from 2 species of *Oryza* genus, including *Oryza sativa Japonica* Group (GenBank: BAD31281, BAD01425, NP_001061476) and *Oryza sativa Indica* Group (GenBank: Np_001067785, BAI39872, BAI39873), were downloaded from NCBI [47]. Sequence alignments were performed using the CLUSTAL W program set in MEGA 5.0 program (Tamura, Tokyo, Japan). Phylogenetic analyses and statistical neighbour-joining bootstrap tests of the phylogenies in which the Bootstrap number is 200 were carried out using the MEGA package.

## 4. Conclusions

The present study used *de novo* transcriptome analysis to determine the disease-resistance genes in the three tissues (*i.e.*, leaves, stems and roots) of *O. officinalis*, an important wild species in the *Oryza* genus with high resistance to many diseases. Through *de novo* assembly, 137,229 contigs were obtained ranging from 200 to 19,214 bp with an N50 of 2331 bp using Illumina HiSeq 2000 platform. A total of 88,249 contigs were annotated with gene descriptions by sequence similarity searching against a non-redundant protein database, and 75,589 transcripts were further assigned to GO terms. Based on the KEGG pathway assignments, we identified 22 contigs involved in plant–pathogen interaction, which includes PAMP-triggered immunity and effector-triggered immunity. The results from expression analysis of genes related to plant–pathogen interaction in different tissues showed that the majority of genes were all expressed in the three tissues. Candidate genes for plant hormone regulation pathways were also identified. In addition, two kinds of rice bacterial blight-resistant genes in *O. officinalis*, including two Xa1 genes and three Xa26 genes, were also identified. Both Xa1 genes showed the highest expression level in the stem, whereas one Xa26 gene was expressed dominantly in leaves and another Xa26 genes displayed low expression level in all three tissues. These transcriptome data have led to the identification of a number of candidate genes and functional elements determining plant–pathogen interaction and plant hormone regulation pathways associated with disease resistance, which will accelerate functional genomic studies of *O. officinalis* and genetic improvement efforts for cultivated rice in the future.

## Supplementary Materials

Supplementary Materials are available online at www.mdpi.com/1422-0067/16/12/26178/s1.

## Availability of Supporting Data

The full data sets of *O. officinalis* leaves, stems and roots have been submitted to NCBI SRA databases under Accession SRR2102055, SRR2102056 and SRR2102057.
